# Health-related quality of life in children with congenital heart disease following interventional closure versus minimally invasive closure

**DOI:** 10.3389/fcvm.2022.974720

**Published:** 2022-10-06

**Authors:** Yuxing Yuan, Bo Pan, Xiaohua Liang, Tiewei Lv, Jie Tian

**Affiliations:** ^1^Department of Cardiology, Ministry of Education Key Laboratory of Child Development and Disorders, National Clinical Research Center for Child Health and Disorders, China International Science and Technology Cooperation Base of Child Development and Critical Disorders, Children’s Hospital of Chongqing Medical University, Chongqing, China; ^2^Chongqing Key Laboratory of Pediatrics, Chongqing, China

**Keywords:** health-related quality of life, minimally invasive closure, interventional closure, ventricular septal defect, atrial septal defect

## Abstract

**Introduction:**

The presence of atrial septal defect (ASD) or ventricular septal defect (VSD) significantly affects children’s quality of life and, if not treated adequately, can contribute to increased mortality. In this study, we evaluated and compared the health-related quality of life (HRQL) of children who underwent treatment using either minimally invasive closure (MIC) or interventional closure (IC).

**Materials and methods:**

In this observational and comparative study 199 children (2 to 4.5 years of age) underwent closure treatment for simple ASD or VSD at the Children’s Hospital of Chongqing Medical University between February 2021 and September 2021. Of these, 116 were treated with IC and 83 with MIC. Both preoperative and postoperative HRQL scores were assessed using the PedsQL^TM3.0^ Cardiac Module and the children were followed up at 3 and 6 months after surgery.

**Results:**

The two groups did not differ significantly in terms of demographics, baseline clinical characteristics, or pre-operative data. The duration of anesthesia (45 mins vs. 109 mins), procedures (25 mins vs. 48 mins), and length of postoperative hospital stay (4.32 days vs. 6.87 days) in the IC group were significantly less than in the MIC group (*P* < 0.001). The incidence of postoperative pneumonia in the VSD patients who underwent MIC was significantly higher than in those who underwent IC treatment (28.9% vs. 0 percent, *P* < 0.001). The HRQL scores increased significantly in both groups following treatment and follow-up evaluations (*P* < 0.001). The mean HRQL score of the IC group 3 months after treatment was significantly higher than that of the MIC group (88.9 vs. 85.7, *P* < 0.001), indicating a significant increase from the baseline score compared with the MIC group (5.4 vs. 2.6, *P* < 0.001). The IC group also showed higher scores than the MIC group (*P* < 0.05) in the dimensions of “Heart Problems and Treatment,” “Treatment Anxiety,” and “Cognitive Problems,” with higher scores indicating fewer problems.

**Conclusion:**

The health-related quality of life in children with ASD and VSD improved continuously regardless of IC or MIC intervention. However, IC led to better HRQL in the early postoperative stage.

## Plain language summary

Congenital heart disease (CHD) is the most frequent birth defect. Although a vast majority of CHD children can survive to adulthood without any cardiovascular events, the optimum treatment for children with simple CHD, such as atrial septal defect (ASD) and ventricular septal defect (VSD), remains contentious. This study aimed to evaluate and compare health-related quality of life (HRQL) in CHD children who underwent either interventional closure (IC) or minimally invasive closure (MIC) therapy. A total of 199 children’s data were collected from the IC group (*n* = 116) and the MIC group (*n* = 83). Demographics, baseline clinical characteristics, and preoperative data were generally balanced between the two groups. All patients were assessed for preoperative and postoperative HRQL scores and subsequent follow-ups at 3 and 6 months. The results showed that the HRQL in CHD children improved continuously regardless of the type of treatment (MIC or IC). However, IC could lead to better HRQL in the early postoperative stage.

## Introduction

Congenital heart disease (CHD) is the most common congenital malformation, originating from abnormal or dysfunctional fetal development of the cardiovascular system (1). With improvements in the diagnosis and treatment of CHD, a vast majority of children survive to adulthood without any cardiovascular event (2, 3). Therefore, the health-related quality of life (HRQL) of CHD patients after treatment has attracted the attention of more and more researchers (4).

The HRQL forms an important part of the evaluation of children with CHD as it is not only a predictive index of mortality and hospitalization rate but also a reference for the allocation of future medical resources (5, 6). As an emerging medical evaluation indicator, HRQL was recommended by the Centers for Disease Control and Prevention (CDC) and the American Heart Association (AHA) for the post-treatment evaluation of CHD (7, 8). Previous reports have shown that the HRQL correlated with the type of treatment received by CHD patients (9). Compared with interventional closure (IC), traditional open-chest surgery under cardiopulmonary bypass has several disadvantages including higher levels of surgical trauma, long surgery time, slow postoperative recovery, and scarring, which tend to reduce the children’s HRQL after surgery (10). Minimally invasive closure (MIC) might bring about great improvement. This is a novel surgical technique performed under the guidance of transesophageal bypass with minimal surgical trauma. It is currently increasingly used for the treatment of ventricular septal defect (VSD) and atrial septal defect (ASD) (11–14). In this investigation, we hypothesized that IC results in better postoperative HRQL in children with VSD or ASD than MIC. A professional scale assessment was used for verifying the hypothesis.

## Materials and methods

The study protocol was approved by the Ethical Committee of Chongqing Medical University. Informed consent was provided by the parents of all participants. The research data were kept confidential.

### Patients

#### Inclusion criteria

1. Children diagnosed with VSD or ASD by transthoracic echocardiography before procedures.
2. Children aged 2–4.5 years.

#### Exclusion criteria

**FIGURE 1:**
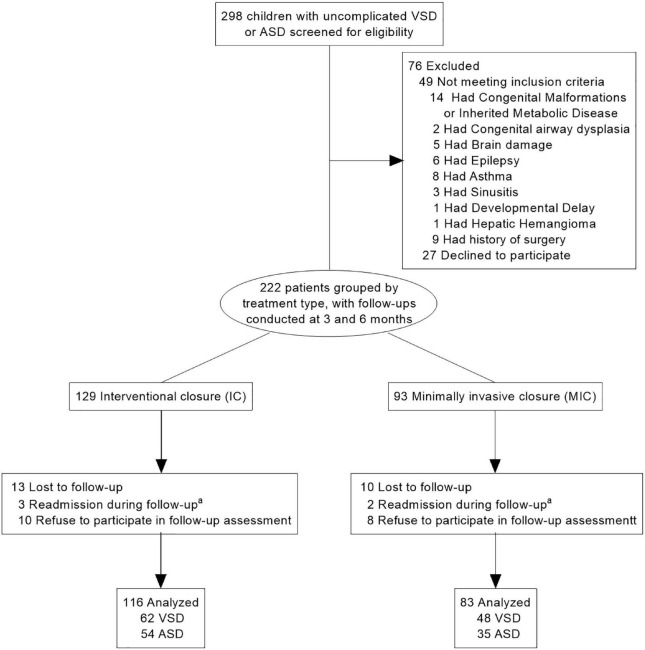
Participant flow in the clinical study. VSD, ventricular septal defect; ASD, atrial septal defect. ^a^Including pneumonia and infectious diarrhea, one person each.

1. Patients with concurrent complicated CHD.
2. Patients with concurrent cognitive impairment, other congenital developmental abnormalities, or chronic disorders (Figure 1).

3. Patients with a history of previous surgery.
4. Patients re-admitted for other medical conditions during the follow-up period.

### Treatment

IC therapy was performed with X-ray assistance (TEE was used to supplement X-ray imaging for catheter guidance and intraoperative evaluation of the procedures), which allows the visualization of the entire procedure, offers the advantages of advanced technology, produces minimal trauma, and allows fast recovery. In contrast, MIC was performed only with TEE assistance and has the advantages of wide indications and no radiation. The procedures were performed under intravenous-inhalation compound anesthesia with tracheal intubation in all patients.

For ASD, the paths for both MIC and IC were the same, with catheter access from the right femoral vein, passing through the inferior vena cava → right atrium → ASD → left atrium → left pulmonary vein. Since MIC had no X-ray assistance, the entire length of the catheter, guide wire, sheath, and the extent of the guide wire entering the pulmonary vein were not visible, potentially causing damage to the pulmonary vein wall (13, 15).

For VSD, the IC was cannulated through the femoral artery, through the aorta → left ventricle → VSD → right ventricle → inferior vena cava, after which the femoral vein approach was used to capture the wire and establish the arteriovenous circuit. During MIC, a small incision of 2–4 cm in the chest was made, the pericardium was cut and suspended, and TEE was used to guide the positioning of the right ventricular surface purse string suture. The right ventricular wall was then punctured and the occlusion was completed through the right ventricle → VSD → left ventricle. The MIC allows a better depiction of the heart’s anatomy without the restriction of vascular conditions; however, it causes more pain and trauma than IC (14, 16, 17).

All children were given aspirin for 6 months post-surgery. Additionally, as per the clinical rule of our hospital, antibiotics and non-invasive ventilator support were given for 24 h postoperatively in children that received MIC.

### Study design

This observational and comparative investigation was carried out from February 2021 to March 2022 (patients from February 2021 to September 2021 were included, with 6-months follow-up). All patients underwent treatment for CHD at the Children’s Hospital of Chongqing Medical University. Patients were divided into two groups according to the treatments they received, namely, the MIC and IC groups. HRQL scores were assessed preoperatively and at the 3- and 6-month follow-ups. The preoperative evaluation was conducted in the ward, and the questionnaire was completed with the guidance of the patient’s caregiver. The postoperative assessment was done by phone or via WeChat.

### Health-related quality of life score

The children’s HRQL was assessed using The Pediatric Quality of life Inventory Measurement Models™ (PedsQL™). The PedsQL™ was developed in 1987 by Professor Varni and his team in the USA and includes general-purpose and disease-specific modules and has shown good reliability and validity. The PedsQL^TM3.0^ Cardiac Module is widely used to assess the HRQL of children with heart disease (5, 6, 10).

The Chinese Version of the PedsQL^TM3.0^ Cardiac Module was translated and culturally adapted by a research group from Sun Yat-sen University, China, under international guidelines (18). The psychometric properties of the Chinese Version PedsQL ^TM3.0^ show good applicability (19, 20). The questionnaire has five dimensions, namely, Heart Problems and Treatment (7 + 3 items), Perceived Physical Appearance (3 items), Treatment Anxiety (4 items), Cognitive Problems (3 items), and Communication (3 items). Each item uses a 5-point Likert scale ranging from 0 to 4 (0, never a problem; 1, rarely a problem; 2, sometimes a problem; 3, often a problem; 4, almost always a problem). Items were reverse-scored and linearly transformed to a 0–100 scale, with higher scores indicating a better HRQL.

### Assessments

We collected the following patient’s information from the hospital’s electronic medical records and the family using a questionnaire:

1. Basic information: sex, age, height, weight, address, caregiver and education, family income, and medical expenses.
2. Preoperative data: clinical symptoms, Modified Ross Score, defect type and size, left ventricular ejection fraction (LVEF), and left ventricular shortening fraction (LVFS).
3. Procedure-related information: duration of anesthesia and procedures, intra- or postoperative bleeding, postoperative hospital stay, and adverse events.
4. Follow-up data: symptoms and examination results at the time of outpatient follow-up consultation, etc.

H Height and weight were expressed in standard deviation units (*Z*-scores) for children of the same age and sex (21). Medical expenses included medical insurance coverage and the full payment covered by self-paying patients. Clinical symptoms mainly included hyperhidrosis, shortness of breath or cyanosis after activity, and recurrent respiratory infections. The Modified Ross Score was used to rate the children’s cardiac function through history and physical examination. VSD defects were divided into peri-membranous, sub arterial, and muscular defects while ASD defects were divided into ostium primum, ostium secundum, and coronary sinus defects; all the ASD children included in the study had the secundum type. Postoperative adverse events were evaluated according to international standards (22) and included the new onset of moderate or severe heart valve reflux, pericardial effusion, and other complications.

Transthoracic Doppler echocardiography and electrocardiograms were routinely performed for 3–7 days after surgery. In addition, transthoracic Doppler echocardiography and X-rays were performed during the follow-ups at 3 and 6 months post-surgery. All patients received oral aspirin 3–5 mg/kg daily for 3–6 months.

### Endpoints

The primary endpoint was the HRQL score 3 months after the procedure. Secondary endpoints were the HRQL score 6 months after the procedure, the complications encountered, and the cardiac ultrasound and electrocardiogram results.

### Statistical analysis

The number of patients required for this study was computed using the software G*power 3.1. The sample size calculation was based on the results of pre-experimentation, where 30 children with ASD or VSD treated with IC and MIC were randomly selected and followed up for 3 months post-surgery, resulting in a 3-month HRQL score of 90.00 ± 5.83 in the IC group and a score of 85.27 ± 8.38 in the MIC group. A one-sided 0.05 level of significance and a sample size of 80 patients (40 per group) provided 90% statistical efficiency to demonstrate the difference in HRQL score 3 months after surgery. To accommodate for a 15% attrition rate, we had to recruit a total of 96 patients (G*power 3.1, independent tests; allocation ration-1).

The Kolmogorov–Smirnov test was applied to verify whether our data were normally distributed. Means and standard deviations were calculated to describe the variables for normally distributed data, while for non-normally distributed data, medians and ranges were calculated. Frequencies and percentages were used for categorical data. Significant differences between the two groups were measured using χ^2^ or Fischer exact tests for categorical variables and the *t*-test or Wilcoxon rank-sum test for continuous variables. To compare the postoperative HRQL scores between the MIC and IC groups with adjustment on preoperative scores, a covariance analysis (ANCOVA) was performed. A *P*-value < 0.05 was considered statistically different. All statistical analyses were performed on SPSS version 24 (IBM, Armonk, NY, USA) software. No imputation was performed for missing data.

## Results

### Patients

Two hundred and ninety-eight patients with ASD and VSD were initially enrolled during the study period; of these, 222 met the inclusion criteria (129 underwent IC and 93 underwent MIC). After excluding patients who were not followed up for both points of evaluation, 199 patients (89.6%) were finally included (Figure 1). Their guardians and professional doctors determined the treatment for all children.

The study included 93 (46.7%) boys and 106 (53.2%) girls, and their median age was 37 months, 38 months for the boys, and 37 months for the girls. The patients had similar average heights (184/199, 92.5%) and weights (176/199, 88.4%) compared with healthy children of the same age and sex. One hundred and eighty-four patients (92.5%) were under direct parental care. The demographics and baseline characteristics were generally balanced between the two groups (Table 1).

**TABLE 1:** Patient demographics and baseline characteristics.

Parameter	IC (116)	MIC (83)	*P*-value
**Sex, No. (%)**			
Boy	56(48.3)	37(44.6)	
Girls	60(51.7)	46(55.4)	0.61
Age, M (IQR), m	39(30, 47)	37(29, 44)	0.25
**Height, No. (%)^a^**			
>+2SD	7(6.0)	3(3.6)	
−2SD∼+2SD	106(91.4)	78(94.0)	0.73
<−2SD	3(2.6)	2(2.4)	
**Weight, No. (%)^a^**			
>+2SD	12(10.3)	7(8.4)	
−2SD∼+2SD	101(87.1)	75(90.4)	0.69
<−2SD	3(2.6)	1(1.2)	
**Caregiver, No. (%)**			
Parents	108(93.1)	76(91.6)	
Grandparents	8(6.9)	7(8.4)	0.69
**Education, No. (%)^b^**			
Junior and lower	27(23.3)	27(32.5)	
High	40(34.5)	27(32.5)	
Undergraduate	48(41.4)	29(34.9)	0.37
Graduate and above	1(0.9)	0(0.0)	
**Address, No. (%)**			
Village	46(39.7)	28(33.7)	
Town	12(10.3)	11(13.3)	0.64
City	58(50.0)	44(53.0)	
**Expenses, No. (%)^c^**			
Insurance	82(70.7)	57(68.7)	
Self-paying	34(29.3)	26(31.3)	0.76
**Income, No. (%), CNY/m**			
<1,000	4(3.4)	4(4.8)	
1,000∼3,000	10(8.6)	14(16.9)	
3,000∼5,000	42(36.2)	35(42.42)	0.20
5,000∼10,000	49(42.2)	24(28.9)	
>10,000	11(9.5)	6(7.2)	

^a^Height and weight were expressed in standard deviation units (*Z*-scores) with reference to children of the same age and gender.

^b^The level of education of the caregiver.

^c^Medical expenses include medical insurance coverage and full self-paying.

### Preoperative data

The clinical manifestations of participants were mainly hyperhidrosis (82/199, 41.62%), shortness of breath or cyanosis after activity (74/199, 37.2%), and recurrent respiratory tract infection (33/199, 16.6%). The median Modified Ross score was 1 (0, 2). Preoperative cardiac ultrasound showed that 110 patients (55.3%) had VSD (including 102 perimembranous defects, 4 sub-arterial defects, and 4 muscular defects), 89 patients (44.7%) had the ASD secundum type, and the median defect size was 6.7 (4.0, 8.2) mm. Patients had a mean LVEF of 67.15 (4.85)% and a mean LVFS of 36.41 (4.03)%. These features did not differ significantly between the two groups (Table 2).

**TABLE 2:** Patient preoperative data and procedure-related information.

Parameter	IC (116)	MIC (83)	*P*-value
**Clinical, No. (%)^a^**			
Hyperhidrosis	51(44)	31(37.3)	0.35
Shortness of breath or cyanosis after activity	42(36.2)	32(38.6)	0.74
Recurrent respiratory tract infection	17(14.7)	16(19.3)	0.39
Ross, M (IQR)^a^	1(0, 1)	1(0, 2)	0.29
**Defect type, No. (%)^b^**			
**VSD**			
Perimembranous	59(50.9)	43(51.8)	
Subarterial	1(0.9)	3(3.6)	
Muscular	2(1.7)	2(2.4)	0.54
**ASD**			
Secundum	54(46.6)	35(42.2)	
Defect size, M (IQR), mm^b^	62(45,84)	55(40, 80)	0.16
LVEF, mean (SD),%^b^	66.65(4.84)	67.84(4.81)	0.09
LVFS, mean (SD),%^b^	36.17(4.05)	36.75(4.00)	0.32
Duration of anesthesia, M (IQR), min^a^	45(35, 60)	109(80, 126)	<0.001
Duration of procedures, M (IQR), min^a^	25(15, 38)	48(20, 62)	<0.001
Postoperative hospital stay, mean (SD), d^a^	4.32(1.55)	6.87(1.77)	<0.001
Bleeding, No. (%)^a^	0(0.0)	3(3.6)	0.14
**Complications, No. (%)^a^**			
Pneumonia			
VSD	0(0.0)	24(28.9)	<0.001
ASD	0(0.0)	2(6.0)	0.16
Hypoproteinemia	0(0.0)	1(1.2)	0.42
Impaired liver function	2(1.7)	2(2.4)	0.73
Moderate to severe anemia	3(2.6)	0(0.0)	0.38
Tricuspid valve reflux	1(0.9)	5(6.0)	0.61
Aortic valve reflux	1(0.9)	0(0.0)	
Pericardial effusion	0(0.0)	1(1.2)	
**Residual shunt, No. (%)^b^**			
<1 mm	97(83.6)	73(88.0)	0.56
1–2 mm	7(6.0)	5(6.0)	
2–3 mm	12(10.3)	5(6.0)	

None of our subjects developed complete atrioventricular block (CAVB).

^a^Determined by patient-reported medical history and physical examination at the time of admission.

^b^Determined by transthoracic cardiac color Doppler ultrasound.

### Procedure-related information

As Table 2 indicates, both groups had similar bleeding rates and residual shunting. The duration of anesthesia in the IC and MIC groups was 45 mins and 109 mins, respectively, the procedure length was 25 min and 48 min, respectively, and the length of postoperative hospital stay was 4.32 and 6.87 days, respectively, indicating that all these parameters in the IC group were significantly lower compared with the MIC group (*P* < 0.001). The postoperative complications observed were pneumonia, hypoproteinemia, impaired liver function, moderate to severe anemia, and new onset of moderate or severe valve reflux, all of which were diagnosed by clinical presentation and adjunctive tests. Among these, the incidence of postoperative pneumonia in VSD patients treated with the MIC was much higher than in those who underwent IC therapy (28.9% vs. 0 percent, *P* < 0.001).

### Health-related quality of life

**FIGURE 2:**
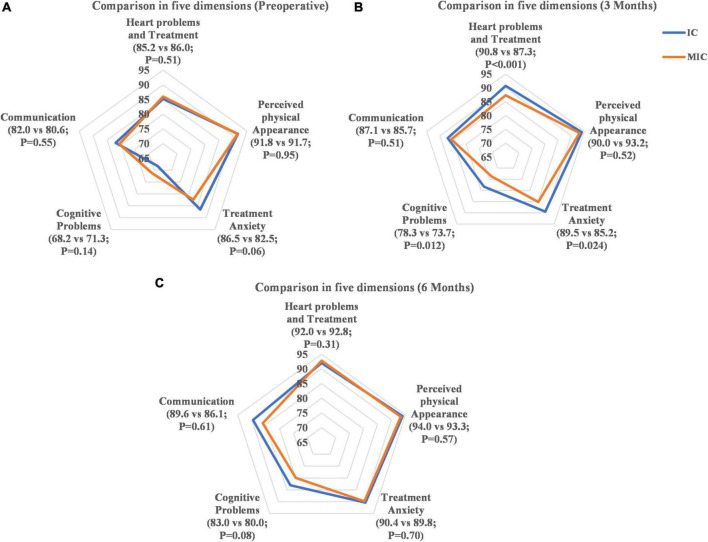
Comparison of dimension scores. **(A)** Comparison in five dimensions (preoperative). **(B)** Comparison in five dimensions (3 months). **(C)** Comparison in five dimensions (6 months).

In accordance with the mean comparison, the total HRQL scores increased significantly in both groups after septal closure procedures and follow-up evaluations at 3 and 6 months (*P* < 0.001). The dimension’s scores of “Cardiac Problems and Treatment,” “Treatment Anxiety,” “Cognitive Problems,” and “Communication” increased significantly in both groups after 6 months (*P* < 0.05) (Figure 2). There was no significant difference in the HRQL scores (total and five dimensions) between the two groups at the preoperative and 6-month follow-up assessments.

**FIGURE 3:**
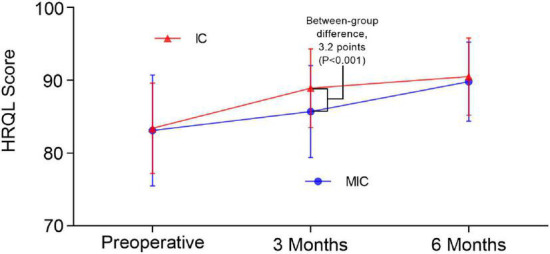
Mean HRQL over time (error bars represent SD). *The inter-group comparison used independent-samples *t*-test. Comparison over time used paired-samples *t*-test. a. Preoperative: IC group, 83.4(6.2), MIC group 83.1(7.6), *P* = 0.75. b. 3 months: IC group, 88.9(5.4), MIC group 85.7(6.3), *P* < 0.001. c. 6 months: IC group, 90.5(5.3), MIC group 89.8(5.4), *P* = 0.36. d. Compared to preoperatively, both groups had *P* < 0.001 at 3 and 6 months.

The HRQL score of the IC group at the 3-month follow-up was significantly higher than that in the MIC group (88.9 vs. 85.7, respectively, *P* < 0.001) (Figure 3). Three months after closure therapy, the HRQL score of the IC group had increased by 5.4 (6.5) points, while that of the MIC group had increased by only 2.6 (5.3) points (difference, 2.99 [95% CI 1.62–4.37]; *P* < 0.001); for VSD, the IC group increased by 5.7 (7.1) points, while the MIC group increased by only 3.1 (5.1) points (difference, 3.27 [95% CI 1.42–5.31]; *P* = 0.002); for ASD, the IC group increased by 5.1 (5.9) points, and the MIC group increased by only 1.9 (5.7) points (difference, 2.71 [95% CI 0.86–4.56]; *P* = 0.002; Table 3). The IC group also showed higher scores than the MIC group (*P* < 0.05) in the dimensions of “Heart Problems and Treatment,” “Treatment Anxiety,” and “Cognitive Problems” (Figure 2).

**TABLE 3:** Changes in the health-related quality of life scores from the preoperative stage.

Time	IC	MIC	Adjusted mean difference (95% CI)*	*P*-value
Total	*N* = 116	*N* = 83		
3 months	5.4(6.5)	2.6(5.3)	2.99(1.62–4.37)	<0.001
6 months	7.1(7.1)	6.7(6.7)	0.61(−0.79–2.02)	0.39
VSD	*N* = 62	*N* = 48		
3 months	5.7(7.1)	3.1(5.1)	3.27(1.24–5.31)	0.002
6 months	7.4(7.0)	7.4(5.9)	0.80(−1.14–2.74)	0.41
ASD	*N* = 54	*N* = 35		
3 months	5.1(5.9)	1.9(5.7)	2.71(0.86–4.56)	0.005
6 months	6.7(7.3)	5.7(7.8)	0.30(−1.79–2.38)	0.78

*Adjusted preoperative scores.

## Discussion

Currently, most of the studies on HRQL associated with CHD children have focused on evaluating the differences and causes of HRQL scores between CHD and healthy children. Several studies have concluded that children with CHD have lower HRQL than healthy children (17, 23, 24); however, some reports have contradicted these results (6, 25, 26), possibly due to the variety of influencing factors such as the degree of national development, socioeconomic level, underlying medical conditions, and parental education. In the past, CHD treatments were evaluated mainly from the perspective of improvement in physiological function. However, with advances in current medical technology, it has become important to compare the impact of different treatment strategies on the children’s HRQL scores.

The literature suggests that severe CHD affects physical, cognitive, and neurological development which, in turn, affects the HRQL of children (27, 28). In our study, all children underwent successful septal defect repairs, and only 17 (8.5%) had postoperative residual shunts of 2–3 mm, which either closed spontaneously or were < 2 mm 6 months after surgery. Importantly, the children’s HRQL scores were all significantly higher than the preoperative scores. Therefore, these two treatment strategies can effectively improve the children’s hemodynamic and cardiac function defects resulting in the improvement of the HRQL in children with CHD.

In terms of treatment, we observed that the durations of anesthesia and procedures during MIC were much longer than with IC. Moreover, in VSD patients, although the MIC incision was smaller than that used in traditional surgery, there was still more postoperative pain and scarring compared with IC. Additionally, the use of antibiotics and non-invasive ventilators may also increase the child’s adverse experience. Altogether, these factors can prolong the length of hospitalization as well as increase a child’s fear, anxiety, and other negative emotions following the surgery, thus, ultimately reducing the HRQL in the early postoperative period.

Recent studies have identified complete atrioventricular block (CAVB) as a complication associated with interventional or surgical therapy in CHD. The incidence of CAVB is 0.9–1.6% in MIC, which is lower compared with IC (1.0–5.0%) (29–32). None of our subjects, however, developed CAVB, which might be due to our small sample size. In our investigation, children with VSD who underwent MIC had a higher incidence of pneumonia, possibly associated with the procedure path through the chest incision, which is also a major factor contributing to prolonged postoperative hospital stay resulting in increased anxiety in both children and their families and thus affecting the quality of life in the early postoperative stage.

Considering the scores of each dimension, our results showed that the “Perceived Physical Appearance” scores were relatively higher in both the preoperative and postoperative data (compared with other dimensions), with no significant difference between the groups. We considered this to be associated with the fact that the children were younger and had fewer negative feelings about their appearance.

The dimension of “Heart Problems and Treatment” reflects mainly physiological changes. The occlusion therapy effectively corrected the children’s cardiac malformations and improved the abnormal hemodynamics, thus reducing and gradually eliminating the heart-related symptoms and leading to improvements in the children’s diet, activity, and growth and development. This would account for the improvement in the scores for this dimension.

The “Treatment Anxiety” and “Cognitive Problems” categories reflect alterations in children’s psychological domains. Due to the influence of the CHD itself, these children had extensive experience of medical environments even before the surgery, leading to more medical-associated fears. Furthermore, a sense of uncertainty about the child’s circumstances often causes anxiety and depression in parents, and these negative emotions indirectly affect the child’s physical, emotional, and social functioning. This anxiety in parents can lead to overprotection of the children, reducing the children’s opportunities for contact and communication with the outside world and thus affecting their social and psychological adaptability, thereby reducing the HRQL levels (33, 34). After occlusion, with the alleviation of the disease, the outlook of both parents and children tended to improve, creating a healthy and positive environment. This can significantly improve the scores in the postoperative psychological field (35). Increasing age is also associated with improved cognitive development, thereby affecting score levels. Similarly, with the increase in age and the change in the family atmosphere, the children’s ability to communicate, together with their improved environment and conditions, also affects the score of the “Communication” dimension. In the early postoperative period, the scores of “Heart problems and Treatment,” “Treatment Anxiety,” and “Cognitive Problems” in the IC group were significantly higher than those in the MIC group, likely associated with the aforementioned factors; furthermore, in case of MIC, family members may be more anxious about the surgery, leading to more negative emotions, which may indirectly affect the children’s scores in this field.

In conclusion, MIC was associated with a longer duration of anesthesia, procedure, hospital stay, higher incidence of pneumonia, and postoperative pain and scarring. All these factors increased the children’s negative emotions and bad memories during hospitalization, thus lowering their HRQL in the early postoperative period. Moreover, for the family members, their uncertainty surrounding the early postoperative efficacy, anxiety associated with postoperative complications, and the psychological burden of the treatment procedure affect the family’s attitude toward the children, thus indirectly influencing their HRQL during the early postoperative period. Therefore, we believe that IC can offer a better HRQL in the early postoperative stage than MIC. On the other hand, if better psychological support is provided for children and their families at this stage, the HRQL scores could be improved.

It was also interesting that by 6 months, the children had gradually forgotten the bad memories and negative emotions associated with the treatment. The parents also showed more positive attitudes toward the postoperative curative effect, so there was no significant difference in HRQL scores between the two groups 6 months after the procedure.

## Limitations

As the sample size was restricted due to the children’s age and length of study, the study only represents a small sample of the VSD and ASD population in China. Multi-center studies with larger sample sizes, multiple ages, and types of CHD are warranted to offer a better representation and to further substantiate our findings. The study also lacks the normal reference value of HRQL of healthy children in China, and further studies will be carried out to support this data.

## Conclusion

The study observed that the HRQL in children with CHD of the VSD or ASD secundum types showed continuous improvement after treatment, irrespective of whether IC or MIC was used. However, IC treatment was found to lead to a better HRQL in the early postoperative stage, as it involved shorter anesthesia and procedure duration, which significantly minimized complications and improved the treatment results.

## Data availability statement

The data that support the findings of this study are not publicly available due to privacy or ethical restrictions. Requests to access these datasets should be directed to YY, 978773131@qq.com.

## Ethics statement

The studies involving human participants were reviewed and approved by Ethical Committee of Chongqing Medical University. Written informed consent to participate in this study was provided by the participants’ legal guardian/next of kin. Written informed consent was obtained from the minor(s)’ legal guardian/next of kin for the publication of any potentially identifiable images or data included in this article.

## Author contributions

YY, BP, and XL were performed by material preparation, data collection, and analysis. YY wrote the first draft of the manuscript. All authors contributed to the study conception and design, commented on previous versions of the manuscript, and read and approved the final manuscript.
