# Supplementary figures and images for: Health-related quality of life in children with congenital heart disease following interventional closure versus minimally invasive closure
Source: Front Cardiovasc Med. 2022 Oct 6;9:974720. doi: 10.3389/fcvm.2022.974720 (PMC9583532; doi:10.3389/fcvm.2022.974720)

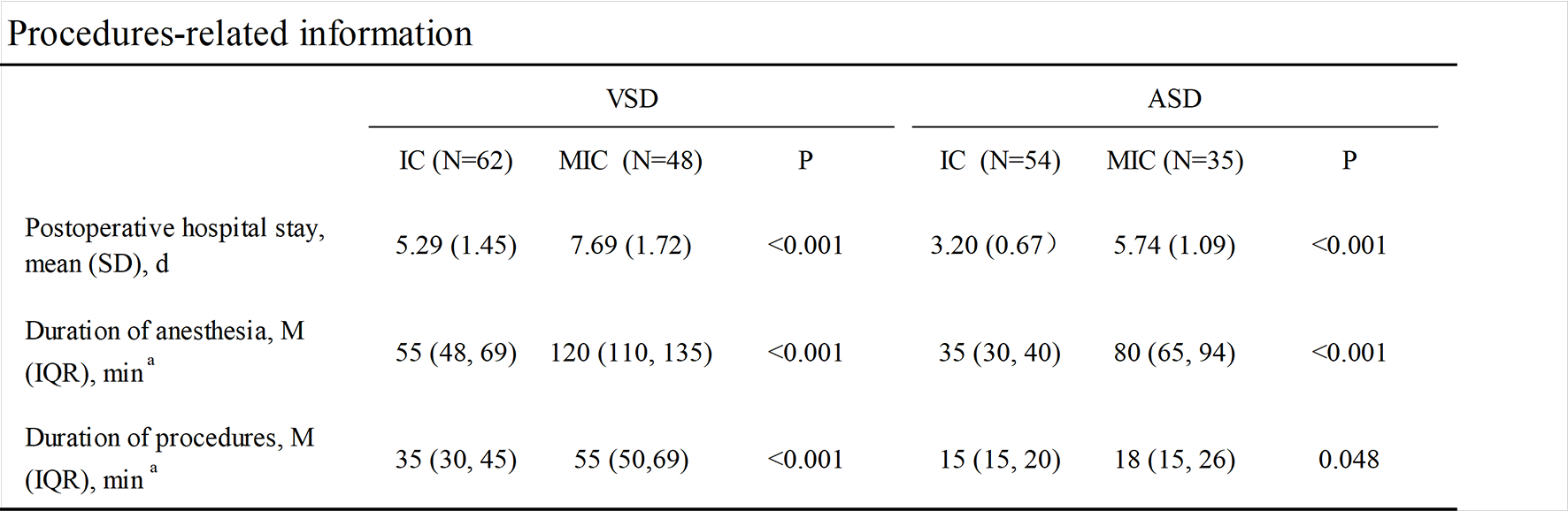

Supplement: Supplementary file 1 [file Image_1.TIF]
